# *FAIM* Is Regulated by MiR-206, MiR-1-3p and MiR-133b

**DOI:** 10.3389/fcell.2020.584606

**Published:** 2020-12-23

**Authors:** Elena Coccia, Marc Masanas, Joaquín López-Soriano, Miguel F. Segura, Joan X. Comella, M. José Pérez-García

**Affiliations:** ^1^Cell Signaling and Apoptosis Group, Vall d’Hebron Research Institute, Barcelona, Spain; ^2^Centro de Investigación Biomédica en Red sobre Enfermedades Neurodegenerativas (CIBERNED), Madrid, Spain; ^3^Institut de Neurociències, Departament de Bioquímica i Biologia Molecular, Facultat de Medicina, Universitat Autònoma de Barcelona, Bellaterra, Spain; ^4^Group of Translational Research in Child and Adolescent Cancer, Vall d’Hebron Research Institute (VHIR)-UAB, Barcelona, Spain

**Keywords:** microRNA, neurodegenerative diseases, nervous system, death receptor, FAIM, Fas apoptotic inhibitory molecule

## Abstract

Apoptosis plays an important role during development, control of tissue homeostasis and in pathological contexts. Apoptosis is executed mainly through the intrinsic pathway or the death receptor pathway, i.e., extrinsic pathway. These processes are tightly controlled by positive and negative regulators that dictate pro- or anti-apoptotic death receptor signaling. One of these regulators is the Fas Apoptotic Inhibitory Molecule (FAIM). This death receptor antagonist has two main isoforms, FAIM-S (short) which is the ubiquitously expressed, and a longer isoform, FAIM-L (long), which is mainly expressed in the nervous system. Despite its role as a death receptor antagonist, FAIM also participates in cell death-independent processes such as nerve growth factor-induced neuritogenesis or synaptic transmission. Moreover, FAIM isoforms have been implicated in blocking the formation of protein aggregates under stress conditions or de-regulated in certain pathologies such as Alzheimer’s and Parkinson’s diseases. Despite the role of FAIM in physiological and pathological processes, little is known about the molecular mechanisms involved in the regulation of its expression. Here, we seek to investigate the post-transcriptional regulation of FAIM isoforms by microRNAs (miRNAs). We found that miR-206, miR-1-3p, and miR-133b are direct regulators of FAIM expression. These findings provide new insights into the regulation of FAIM and may provide new opportunities for therapeutic intervention in diseases in which the expression of FAIM is altered.

## Introduction

Several types of molecules are able to block apoptotic pathways, conferring cells with protection against threatening stimuli. The extrinsic apoptotic pathway is mediated by death receptors that integrate and transmit the extracellular apoptotic stimuli. In the last 20 years, mounting evidence has shed light on the physiological and pathological functions of these molecules and has widened the array of identified responses elicited by these receptors beyond cell death. Indeed, Fas receptor and TNF receptors (TNFRs) are paradigmatic cases of receptors that can trigger apoptotic and non-apoptotic responses depending on the cellular and molecular context (Marques-Fernandez et al., 2013).

The molecular response upon death receptor activation, depends on the activity of proteins called death receptor antagonists. Among these, FAIM (Fas apoptosis inhibitory molecule) was first identified as a negative regulator of Fas signaling (Schneider et al., 1999). It was later found to play multifaceted roles in other physiological processes such as the protective or deleterious effects of TNFα in neurodegenerative disorders (Carriba et al., 2015), regulating axon-selective pruning, hippocampal long-term depression (LTD) (Martinez-Marmol et al., 2016) and opposition to stress-induced accumulation of protein aggregates (Kaku and Rothstein, 2020).

Two main FAIM isoforms generated by alternative splicing have been found at the protein level. While the shorter isoform, named FAIM-S, is ubiquitously expressed, FAIM-L is expressed exclusively in neurons and testes (Zhong et al., 2001; Segura et al., 2007). In the nervous system, FAIM-S participates in neurite outgrowth by activating Ras-ERK and NF-κB pathways. On the other hand, FAIM-L has been shown to modulate death receptor-induced apoptosis and caspase activation by binding to the receptor (Segura et al., 2007), as well as through interaction with X-linked inhibitor of apoptosis (XIAP) (Moubarak et al., 2013).

Alterations in the expression of FAIM may be relevant in several types of human diseases. For example, in multiple myeloma (MM) patients, FAIM expression is increased in B lymphocyte cells compared with normal individuals and its expression is higher in symptomatic MM patients compared with asymptomatic and premalignant individuals (Huo et al., 2013). *FAIM* expression is also elevated in CD34 hematopoietic stem cells and leukocytes. This deregulation is associated with chronic myeloproliferative pathogenesis (Tognon et al., 2011).

Other results show FAIM as an important molecule in metabolic processes. When both isoforms of FAIM are knocked out, mice spontaneously develop non-hyperphagic obesity, as well as also manifest hepatosteatosis, adipocyte hypertrophy, dyslipidemia, hyperglycemia, and hyperinsulinemia. In obese patients, FAIM expression is lower in blood cells and is inversely correlated with insulin resistance biomarkers (Huo et al., 2016).

Moreover, FAIM-L levels have been found to be relevant in neurodegenerative diseases. FAIM-L was found to be reduced in the hippocampus of Alzheimer’s disease patients (Carriba et al., 2015) and in the entorhinal and hippocampal cortex of Alzheimer’s disease mouse models (APP-PS1) (Carriba et al., 2015). In Parkinson’s disease, the expression of FAIM-L was found to be reduced in midbrain dopaminergic neurons after trophic factor deprivation, as well as to sensitize them to Fas-induced cell death (Yu et al., 2008). Recent findings also show that FAIM could play a role in Amyotrophic Lateral Sclerosis inhibiting the aggregation of mutant SOD1, suggesting that FAIM participates in maintaining cell homeostasis (Kaku and Rothstein, 2020). Kaku et al. (2020) also described that FAIM is recruited to cellular stress-induced ubiquitinated proteins, and the levels of stress-induced protein aggregates are much greater in FAIM-deficient cell lines.

Despite the pathological consequences of FAIM de-regulation, little is known about how its expression is modulated. Kaku et al. reported that murine Faim promoter contains three interferon regulatory factor (IRF) binding sites, and Faim expression is positively regulated through IRF4 in primary B cells (Kaku and Rothstein, 2009). At post-transcriptional level, FAIM can also be regulated by MicroRNAs (miRNAs) (Patron et al., 2012; Santosa et al., 2015). MiRNAs are short non-coding RNA of 18–25 base pairs in length that are involved in the regulation of gene expression at the post-transcriptional level. Mature miRNAs repress gene expression through binding to the 3′UTR of the mRNA with the miRNA seed region, a 6–8 bases located at the 5′ end of the mature miRNA and perfectly complementary to the target mRNA sequence (Mullany et al., 2016), thereby inhibiting mRNA translation or inducing mRNA degradation (Alvarez-Garcia and Miska, 2005; Shingara et al., 2005). Thus far, the evidence of FAIM being regulated by miRNA was reported by Patron and colleagues who showed that miR-133b directly impairs the expression of FAIM, thereby enhancing Fas-induced cell death in HeLa and PC3 cells (Patron et al., 2012).

Owing to the pathological consequences that *FAIM* de-regulation may have for certain human diseases like are those involved in neurodegeneration, we sought to screen for other miRNA that could bind to the FAIM 3′UTR and modulate its expression. Our study identified miR-206, miR-1-3p and miR-133b as direct regulators of FAIM, thereby providing a deeper knowledge on the FAIM regulation mechanisms and opening up new opportunities for therapeutic intervention.

## Materials and Methods

### FAIM 3′UTR Analysis

The miRWalk 2.0 database using five miRNA-target prediction algorithms (miRDB (RRID:SCR_010848), miRWalk (Vlachos et al., 2015), miRanda (Betel et al., 2010), miRMap (RRID:SCR_016508) and TargetScan; version 6.2 (Agarwal et al., 2015) were used for the computational miRNA target prediction analysis. The miRNA target search was restricted to the 3′UTR of FAIM and with a minimum complementarity of 7 nucleotides in the seeding region. Probability distribution of random matches was set at 0.05 (Poisson *p*-value). MiRNAs with *p* ≤ 0.05 predicted by all five algorithms were selected for further analysis.

### Cell Culture and Transfection

SH-SY5Y (Cat# CRL-2266, RRID:CVCL_0019), SK-N-AS (Cat# CRL-2137, RRID:CVCL_1700), HEK293T (Cat# CRL-3216, RRID:CVCL_0063) and HeLa (CLS Cat# 300194/p772_HeLa, RRID:CVCL_0030) cell lines were purchased from American Type Culture Collection (ATCC, Rockville, MD, United States). SH-SY5Y, SK-N-AS and HEK293T were grown in Dulbecco’s modified Eagle’s medium (DMEM, Thermo Fisher Scientific, Waltham, MA, United States) containing 10% fetal bovine serum (HEK293T, SK-N-AS) or 15% fetal bovine serum (SH-SY5Y) (Thermo Fisher Scientific, Waltham, MA, United States). HeLa cells were cultured in Roswell Park Memorial Institute (RPMI) 1640 (Thermo Fisher Scientific) supplemented with 10% fetal bovine serum, sodium pyruvate 1 mM (Thermo Fisher Scientific) and 1% of non-essential amino acids (Thermo Fisher Scientific). All media were supplemented with 100 U/mL penicillin, 100 μg/mL streptomycin (Thermo Fisher Scientific) and 5 μg/mL Plasmocin^TM^ (InvivoGen). Culture conditions were maintained at 37°C in a humidified atmosphere containing 5% CO_2_. For miRNA transfection, SH-SY5Y, SK-N-AS and HeLa were seeded at 6 × 10^5^, 4.5 × 10^5^, and 4 × 10^5^ cells in 60 mm dishes, respectively, and transfected 24 h later with the indicated miRIDIAN microRNA mimic oligonucleotides (25 nM, Dharmacon), GE Healthcare using Lipofectamine 2000 transfection reagent (Thermo Fisher Scientific, Waltham, MA, United States), following the manufacturer’s instructions. Mimic Transfection Control with Dy547 was used as a negative control.

### Luciferase Reporter Assay

Wild type and mutated 3′UTR sequences of FAIM were synthetized using the GeneArt Gene synthesis platform (Thermo Fisher Scientific, Waltham, MA, United States) and cloned into the psiCheck^TM^-2 dual luciferase reporter vector (Promega, C8021). For luciferase assays, HEK293T (Cat# CRL-3216, RRID:CVCL_0063) cells were co-transfected with 50 ng of psiCheck^TM^-2 vectors containing wild type or mutated *FAIM* 3′UTR and 25 nM of the indicated miRNAs, using Lipofectamine 2000 (Invitrogen, Carlsberg, CA, United States), following the manufacturer’s protocol. Luciferase activity was measured 24 h post-transfection using the Dual Luciferase Reporter Assay System (Promega Corporation, Madison, WI, United States). Luminescence was measured in an Appliskan (Thermo Fisher Scientific) microplate reader. Renilla luciferase activity was normalized to corresponding firefly luciferase activity and plotted as a percentage of the control.

### Quantitative Real-Time PCR

Total RNA, including small RNA, was isolated from human cell lines using the miRNeasy Mini Kit (Qiagen) following the manufacturer’s instructions. Equal amounts of RNA (1 μg) were converted to cDNA using the High Capacity RNA-to-cDNA Kit (Applied Biosystems), following the manufacturer’s instructions. The quantitative real-time PCR (RT-qPCR) was performed using TaqMan Universal PCR Master Mix Kit (Thermo Fisher Scientific). Samples were subjected to a PCR amplification protocol using an AB7900HT Real Time PCR System (Thermo Fisher Scientific, Waltham, MA, United States) using the following primers for *FAIM-L* (Hs00992098_m1;Thermo Fisher Scientific, Waltham, MA, United States) and for *FAIM* (Hs00216756_m1; Thermo Fisher Scientific, Waltham, MA, United States). The PCR conditions were: 94°C for 3 min, 40 cycles of 45 s at 94°C, followed by 30 s at 55°C, 72°C for 1 min and 72°C for 10 min. The data were analyzed using the SDS 2.3 software (Thermo Fisher Scientific, Waltham, MA, United States) and normalized using GAPDH as a housekeeping gene. TaqMan MicroRNA Assay (Applied Biosystems) was used to convert miRNA to cDNA for the analysis of mature miRNAs. cDNA was quantified by Taqman Universal Master Mix (Applied Biosystems). MiRNA expression was normalized against RNU-44 small RNA. The reactions were performed in triplicate for each sample and incubated in optical 384-well reaction plates. *FAIM* mRNA expression level was calculated as (Rao et al., 2013).

### Western Blot

Proteins were extracted using SET lysis buffer [10 mM Tris–HCl pH7.4, 150 mM NaCl, 1 mM EDTA and 1% sodium dodecyl sulfate (SDS)] and then quantified using a modified Lowry assay (DC protein assay, Bio-Rad). Equal amounts of protein (30 μg per lane) were separated by 10% sodium dodecyl sulfate polyacrylamide gel (SDS-PAGE) electrophoresis, and then transferred onto a polyvinylidene fluoride membrane (PVDF, Merck Millipore, MA, United States). Membranes were blocked with 5% non-fat milk at room temperature for 1 h and then incubated with the primary antibodies against FAIM (1:1000) (Segura et al., 2007) and α-tubulin (1:10000, Cat# T9026, RRID:AB_477593;Sigma-Aldrich) overnight at 4°C. The membranes were then incubated with horseradish peroxidase-conjugated goat anti-rabbit IgG secondary antibody (1:10000, Cat# AP132, RRID:AB_11214051;Sigma-Aldrich) and anti-mouse (1:20000, Cat# AP124, RRID:AB_92455;Sigma-Aldrich) for 1 h at room temperature. An enhanced chemiluminescence detection System, EZ-ECL detection kit (Biological Industries) was used to develop signals, using α-tubulin as a loading control.

### Statistical Analysis

Statistical analysis was performed using GraphPad Prism 7.0. All data in this study were shown as the mean of three independent experiments ± SEM. Statistical differences in multiple groups were examined by one-way ANOVA followed by Dunnett’s multiple range test. *P* value < 0.05 was considered as statistically significant.

## Results

### Five MiRNAs Are Predicted to Target *FAIM* by Five Different MiRNA-Binding Algorithms

**FIGURE 1:**
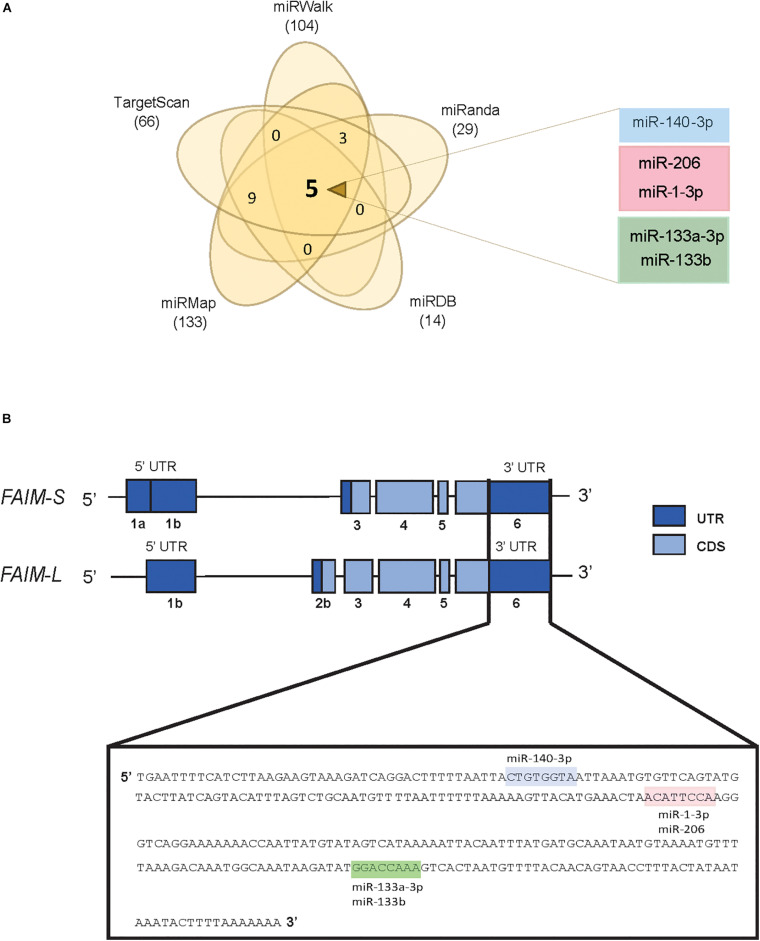
*In silico screening for potential FAIM targeting miRNAs.* **(A)** Venn diagram showing the overlap of potential *FAIM* 3′UTR binding miRNAs using five target prediction tools. **(B)** Schematic representation of the two main FAIM isoforms. Labels shows the miR-140-3p (blue), miR-206/miR-1-3p (pink) and miR-133a-3p/miR133b (green) binding sites in the 3′UTR of *FAIM.* UTR: untranslated region; CDS: coding sequence.

In order to screen for potential miRNAs able to modulate the expression of FAIM, we compared the prediction of putative miRNA-binding sites in the 3′UTR of FAIM from five different prediction algorithms, i.e., TargetScan, miRanda, miRWalk, miRMap, and miRDB (see Supplementary Table 1). When predictions from the different algorithms were overlapped, five miRNAs were commonly found, namely miR-140-3p, miR-206, miR-1-3p, miR-133a-3p, and miR-133b (Figure 1A). The two main isoforms codified by FAIM gene, FAIM-S and FAIM-L, differ in their 5′UTR composition, and in the inclusion of the exon 2b in the neuronal isoform FAIM-L. On the other hand, the 3′ UTR which includes the predicted target sites of these 5 miRNAs, is common to both isoforms (Figure 1B). Of note, while the protein sequence is highly conserved during the evolution, the 3′UTR, and more precisely, the identified miRNA-binding sites are conserved only among vertebrates, thereby suggesting that this mechanism of regulation was incorporated lately in the evolution (Supplementary Figure 1).

### MiR-206, MiR-1-3p and MiR-133b Can Bind Directly to the 3′UTR of FAIM

**FIGURE 2:**
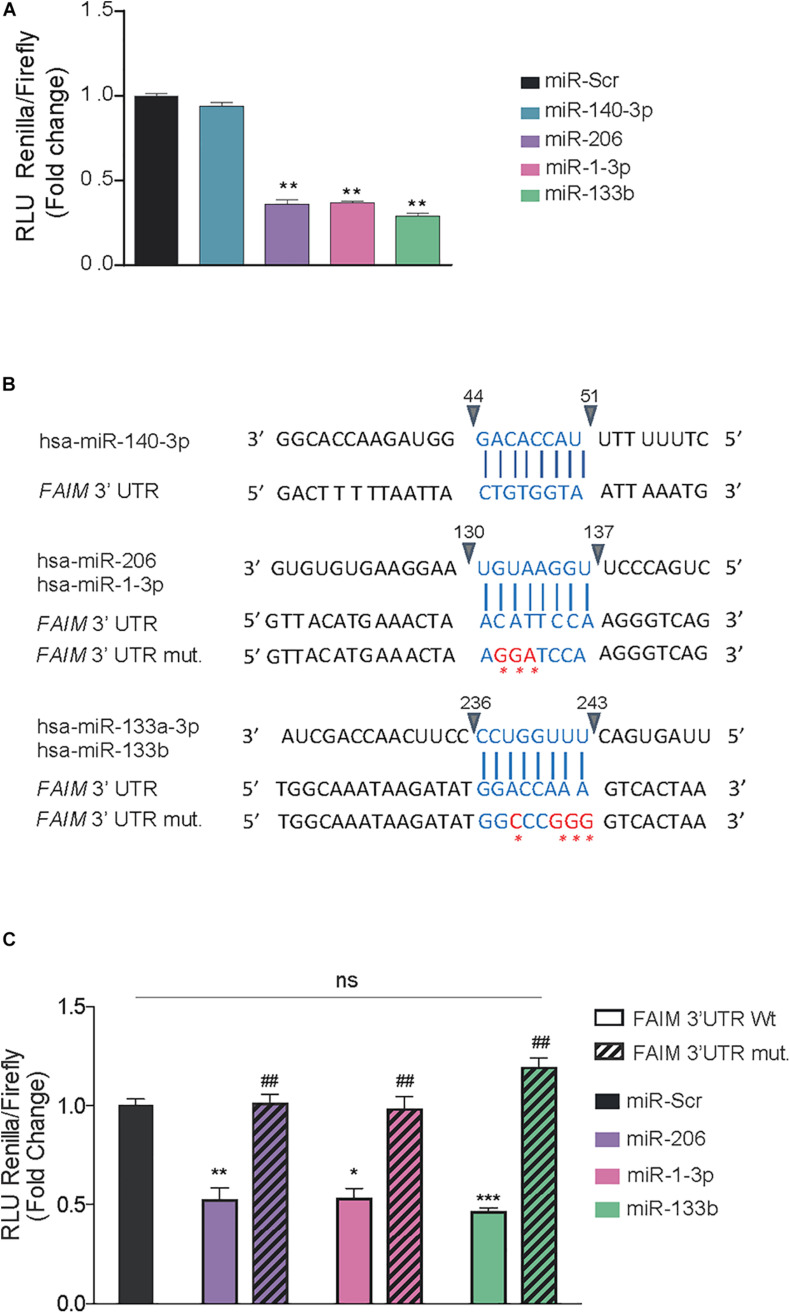
*miR-206, miR-1-3p, and miR-133b bind directly to FAIM 3′UTR*. **(A)** Dual luciferase activity assay in HEK293T cells after the transfection of 25 nM of the indicated miRNAs using psiCheck^TM^-2 vectors encoding the wild type 3′UTR of FAIM. **(B)** Schematic representation of the indicated miRNA binding sites in the wild type (wt) and mutated (mut) 3′UTR of FAIM. The mutated nucleotides are indicated in red. **(C)** Dual luciferase activity assay in HEK293T cells after the transfection of 25 nM of the indicated miRNAs using psiCheck^TM^-2 vectors encoding the wild type and mutated forms of FAIM 3′UTR. Graph represents the values of luciferase activity and are the mean of three independent experiments ± SEM. *P < 0.05, **P < 0.01, and ***P < 0.001, compared with control vector. ##*P* < 0.05 compared with the wild type 3′UTR of FAIM.

To confirm whether the identified miRNAs are truly direct regulators of FAIM expression, a luciferase-reporter vector containing the wild type 3′UTR was cloned and co-transfected with control mimic oligonucleotides or the indicated miRNA mimics. Since miR-133a-3p and miR-133b are almost identical (20/21 nucleotides) and share the exact same seed region, we proceeded with our analyses only with miR-133b. A remarkable reduction in luciferase activity was observed upon transfection of miR-206, miR-1-3p, and miR-133b but not with the transfection of miR-140-3p (Figure 2A). The 3′UTR region of FAIM contains one binding site common to miR-206 and miR-1-3p, and one binding site for miR-133b. In order to confirm the interactions were sequence specific we engineered specific mutations in the 3′UTR, giving rise to correspondingly 3′UTR mut206/1-3p and 3′UTR mut 133b, respectively (Figure 2B). Luciferase activity reduction found in the wild type- 3′UTR was almost completely restored to control levels when miRNAs were co-transfected with the respective 3′UTR mutated forms (Figure 2C). Of note, Clip-seq data mining also showed binding of Ago2 in the 3′UTR of FAIM for miR-133a-3p, miR-133b, miR-206, and miR-1-3p but not for miR-140-3p (Supplementary Table 2). Overall, we were able to show that miR-206, miR-1-3p, and miR-133 have the capacity to directly bind FAIM 3′UTR.

### MiR-206, MiR-1-3p, and MiR-133b Modulate FAIM Expression

**FIGURE 3:**
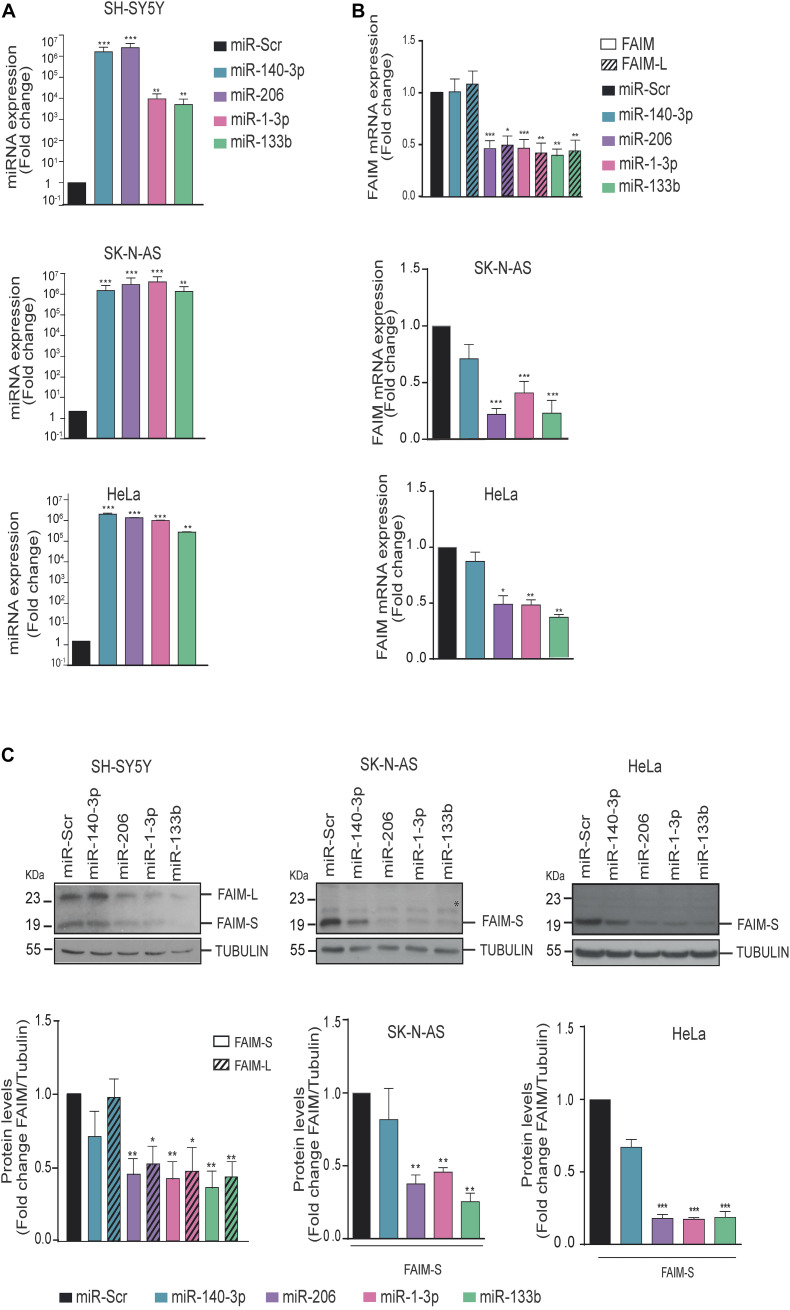
*FAIM expression is modulated by miR-206, miR-1-3p, and miR-133b.* **(A)** MiRNAs expression levels were assessed by RT-qPCR. **(B)** SK-N-AS, SH-SY5Y and HeLa cells were transfected with 25 nM of miR-140-3p, miR-206, miR-1-3p, miR-133b or a control miRNA (miR-Scr) and FAIM mRNA expression levels were assessed by RT-PCR. **(C)** Representative western blots of FAIM in neuroblastoma and HeLa cells transfected with the indicated miRNAs. Lower panels show the quantification of the band intensity of the western blots normalized to the control miRNA (miR-Scr) condition. *Unspecific band. Graphs represent the mean of four independent experiments ± SEM. Tubulin was used as a loading control. *P < 0.05, **P < 0.01, ***P < 0.005.

To elucidate whether the direct binding of miRNAs to the 3′UTR causes a downregulation of FAIM expression, we decided to transfect miRNA mimics oligonucleotides into human cells lines that could represent different tissues where one or both FAIM isoforms are expressed (Figure 3A). On the one hand, we selected the neuroblastoma cell line SH-SY5Y that express both FAIM-L and FAIM-S, and SK-N-AS that only express FAIM-S. Furthermore, we added HeLa cells, since is one of the few models where the functionality of FAIM in human models has been tested (Patron et al., 2012). The expression at mRNA and protein levels was measured in the indicated cell lines after transfection of miR-140-3p, miR-206, miR1-3p, and miR-133b (Figures 3B,C). While miR-140-3p did not modulate the levels of FAIM in any of the cell lines tested, transfection of miR-206, miR-1-3p, and miR-133b caused a ∼2-fold reduction in FAIM mRNA levels (Figure 3B). Similarly, FAIM protein levels decreased in presence of miR-206, miR1-3p, and miR-133b overexpression in the three cell lines tested (Figure 3C). Overall, we were able to confirm that among the predicted miRNAs targeting FAIM 3′UTR, miR-206, miR-1-3p, and miR-133b regulate FAIM isoforms levels, while miR-140 does not.

## Discussion

Death receptor-induced cell death is essential during development due to its role regulating tissue homeostasis and differentiation. In the adult, death receptor signaling can be important under physiological or pathological circumstances. FAIM acts as a death receptor antagonist by binding directly to the death receptor (Segura et al., 2007) or by interacting with downstream effectors such as X-linked inhibitor of apoptosis protein (XIAP) (Moubarak et al., 2013). De-regulation of FAIM is associated with the pathophysiology of cancer and neurodegenerative diseases among others. In Alzheimer’s disease (AD), the levels of *FAIM*-L were shown to be decreased according to Braak stages in AD patients (Braakman et al., 1991; Carriba et al., 2015). At molecular level, FAIM-L levels reduction abolished TNFα protection against amyloid-β neurotoxicity (Carriba et al., 2015). Thus, a better understanding on how FAIM levels are modulated can be paramount for better characterization of human disease and for the design of new therapeutic approaches.

MiRNAs have important roles in regulating diverse biological processes, such as cell proliferation, immunity, development, differentiation, metabolism and cell death, and generally they act as a negative feedback factor in cell signaling (Ha, 2011). Furthermore, miRNA deregulation is a frequent event in human disease, and they can be used as therapeutic tools to treat pathologies with unbalanced cell death and survival pathways (Paul et al., 2018).

We found that miR-206, miR-1-3p, and miR-133b directly regulate FAIM by binding to 3′UTR, decreasing the mRNA and protein levels. MiR-133b has already been described to target FAIM in PC3 and HeLa cells (Patron et al., 2012). The authors showed that *FAIM* silencing or miR-133b overexpression exacerbated death receptor-induced cell death. Our results confirmed that miR-133b is a direct regulator of FAIM in a broader spectrum of cell types including the neuronal lineage. As regards the potential connection of miR-133b-FAIM in neurodegenerative diseases, Jimenez-Jimenez et al. (2014) reported that variations in miR-133b could contribute to the risk of developing Parkinson’s disease. In this regard, the expression of FAIM-L was also described to be reduced in dopaminergic neurons in Parkinson’s disease (Yu et al., 2008), thus making this type of neurons more vulnerable to Fas-induced death. Thus, high levels of miR-133b could contribute to lowering the expression of FAIM in these neurons. However, in Alzheimer’s disease, miR-133b was found to be significantly downregulated after Aβ25-35 treatment (Yang et al., 2019). In a different study, FAIM levels also appear to be reduced in hippocampal samples from AD patients (Carriba et al., 2015), thus suggesting that the miR-133b-FAIM axis would not be relevant in this disease and opens up the question of whether other miRNAs could be responsible for *FAIM* downregulation.

Here, we report, for the first time, that miR-206 and miR-1-3p can also be direct modulators of *FAIM*. Interestingly, miR-206 and miR-1-3p belong to the same miRNA family, which means that, they share the same seed region, thereby suggesting a major overlap in their targets. Furthermore, miR-206, is clustered with miR-133b in the short arm of chromosome 6, indicating that these miRNAs can be co-regulated and provide a strong mechanism in the regulation of *FAIM*. MiR-206 was found to be significantly upregulated in blood samples from Alzheimer’s disease patients compared with age-matched normal controls. Furthermore, upregulation of miR-206 has been detected in serum from patients with mild cognitive impairment (Xie et al., 2015), and in the temporal cortex of human AD brains (Lee et al., 2012). Previous studies using microglial BV-2 cells and miR-206 mimics demonstrated that a pro-inflammatory stimulus (LPS treatment), increased miR-206 expression and enhanced the release of pro-inflammatory cytokines, including IL-1β and TNFα. Thus, in a scenario with high levels of TNFα and low levels of FAIM as reported in some neurodegenerative diseases, TNFα signaling can be switched from a pro-survival to a pro-apoptotic response. Previous results from our lab showing that Aβ treatment decreased the levels of FAIM-L and blocked TNFα protection against Aβ toxicity (Carriba et al., 2015) would support this hypothesis.

To date, there are no effective therapies for these diseases and new strategies are needed. Given the encouraging results of profiling studies and preclinical testing, miRNAs are now being integrated into human clinical trials. For example, miR-122 has successfully reached clinical trials as a targeted therapy for hepatitis C (Lanford et al., 2010). Disrupting the miRNA-mediated reduction of anti-apoptotic proteins such as FAIM, could represent a new neuroprotective strategy against neurodegenerative diseases such as Alzheimer’s or Parkinson’s.

## Data Availability Statement

The raw data supporting the conclusions of this article will be made available by the authors, without undue reservation.

## Author Contributions

EC and MM performed the experiments. MS, JC, and MP-G designed the experiments and wrote the manuscript. EC, MM, MS, and JL-S analyzed data. All authors contributed to the article and approved the submitted version.

## Conflict of Interest

The authors declare that the research was conducted in the absence of any commercial or financial relationships that could be construed as a potential conflict of interest.

## Supplementary Material

Supplementary Figure 1 Multiple sequence alignment of 3′UTR FAIM from different species. Sequences included in the alignment are those of *Homo sapiens*, *Mus musculus*, *Rattus norvegicus*, and *Sus scrofa*. The sequences of miRNA are boxed in blue (miR-140-3p), pink (miR-206;miR-1-3p), and green (miR-133b;miR-133a-3p).

Supplementary Table 1 MiRNA-binding sites in the 3′UTR of FAIM.

Supplementary Table 2 AGO2-ClipSeq studies (Li et al., 2014 and Zhou, ENCORI: the encyclopedia of RNA interactomes).

## References

[B1] Agarwal V. Bell G. W. Nam J. W. Bartel D. P. (2015). Predicting effective microRNA target sites in mammalian mRNAs. *eLife* 4:e05005. 10.7554/eLife.05005 PMC4532895 26267216

[B2] Alvarez-Garcia I. Miska E. A. (2005). MicroRNA functions in animal development and human disease. *Development* 132 4653–4662. 10.1242/dev.02073 16224045

[B3] Betel D. Koppal A. Agius P. Sander C. Leslie C. (2010). Comprehensive modeling of microRNA targets predicts functional non-conserved and non-canonical sites. *Genome Biol.* 11:R90. 10.1186/gb-2010-11-8-r90 PMC2945792 20799968

[B4] Braakman R. Fontijne W. P. Zeegers R. Steenbeek J. R. Tanghe H. L. (1991). Neurological deficit in injuries of the thoracic and lumbar spine. A consecutive series of 70 patients. *Acta Neurochir.* 111 11–17. 10.1007/bf01402507 1927618

[B5] Carriba P. Jimenez S. Navarro V. Moreno-Gonzalez I. Barneda-Zahonero B. Moubarak R. S. (2015). Amyloid-beta reduces the expression of neuronal FAIM-L, thereby shifting the inflammatory response mediated by TNFalpha from neuronal protection to death. *Cell Death Dis.* 6:e1639. 10.1038/cddis.2015.6 25675299 PMC4669818

[B6] Ha T. Y. (2011). The role of MicroRNAs in regulatory T cells and in the immune response. *Immune Netw.* 11 11–41. 10.4110/in.2011.11.1.11 21494372 PMC3072673

[B7] Huo J. Ma Y. Liu J. J. Ho Y. S. Liu S. Soh L. Y. (2016). Loss of Fas apoptosis inhibitory molecule leads to spontaneous obesity and hepatosteatosis. *Cell Death Dis.* 7:e2091. 10.1038/cddis.2016.12 26866272 PMC4849152

[B8] Huo J. Xu S. Lin B. Chng W. J. Lam K. P. (2013). Fas apoptosis inhibitory molecule is upregulated by IGF-1 signaling and modulates Akt activation and IRF4 expression in multiple myeloma. *Leukemia* 27 1165–1171. 10.1038/leu.2012.326 23138182

[B9] Jimenez-Jimenez F. J. Alonso-Navarro H. Garcia-Martin E. Agundez J. A. (2014). Cerebrospinal fluid biochemical studies in patients with Parkinson’s disease: toward a potential search for biomarkers for this disease. *Front. Cell. Neurosci.* 8:369. 10.3389/fncel.2014.00369 25426023 PMC4227512

[B10] Kaku H. Ludlow A. V. Gutknecht M. F. Rothstein T. L. (2020). FAIM opposes aggregation of mutant SOD1 that typifies some forms of familial amyotrophic lateral sclerosis. *Front. Neurosci.* 14:110. 10.3389/fnins.2020.00110 32153351 PMC7047752

[B11] Kaku H. Rothstein T. L. (2009). Fas apoptosis inhibitory molecule expression in B cells is regulated through IRF4 in a feed-forward mechanism. *J. Immunol.* 183 5575–5581. 10.4049/jimmunol.0901988 19843941 PMC3587132

[B12] Kaku H. Rothstein T. L. (2020). FAIM Is a non-redundant defender of cellular viability in the face of heat and oxidative stress and interferes with accumulation of stress-induced protein aggregates. *Front. Mol. Biosci.* 7:32. 10.3389/fmolb.2020.00032 32175331 PMC7056718

[B13] Lanford R. E. Hildebrandt-Eriksen E. S. Petri A. Persson R. Lindow M. Munk M. E. (2010). Therapeutic silencing of microRNA-122 in primates with chronic hepatitis C virus infection. *Science* 327 198–201. 10.1126/science.1178178 19965718 PMC3436126

[B14] Lee S. T. Chu K. Jung K. H. Kim J. H. Huh J. Y. Yoon H. (2012). miR-206 regulates brain-derived neurotrophic factor in Alzheimer disease model. *Ann. Neurol.* 72 269–277. 10.1002/ana.23588 22926857

[B15] Li J. H. Liu S. Zhou H. Qu L. H. Yang J. H. (2014). starBase v2.0: decoding miRNA-ceRNA, miRNA-ncRNA and protein-RNA interaction networks from large-scale CLIP-Seq data. *Nucleic Acids Res.* 42 D92–D97. 24297251 10.1093/nar/gkt1248 PMC3964941

[B16] Marques-Fernandez F. Planells-Ferrer L. Gozzelino R. Galenkamp K. M. Reix S. Llecha-Cano N. (2013). TNFalpha induces survival through the FLIP-L-dependent activation of the MAPK/ERK pathway. *Cell Death Dis.* 4:e493. 10.1038/cddis.2013.25 23412386 PMC3734812

[B17] Martinez-Marmol R. Barneda-Zahonero B. Soto D. Andres R. M. Coccia E. Gasull X. (2016). FAIM-L regulation of XIAP degradation modulates synaptic long-term depression and axon degeneration. *Sci. Rep.* 6:35775. 10.1038/srep35775 PMC5073314 27767058

[B18] Moubarak R. S. Planells-Ferrer L. Urresti J. Reix S. Segura M. F. Carriba P. (2013). FAIM-L is an IAP-binding protein that inhibits XIAP ubiquitinylation and protects from Fas-induced apoptosis. *J. Neurosci.* 33 19262–19275. 10.1523/jneurosci.2479-13.2013 24305822 PMC6618789

[B19] Mullany L. E. Herrick J. S. Wolff R. K. Slattery M. L. (2016). MicroRNA seed region length impact on target messenger RNA expression and survival in colorectal cancer. *PLoS One* 11:e0154177. 10.1371/journal.pone.0154177 27123865 PMC4849741

[B20] Patron J. P. Fendler A. Bild M. Jung U. Muller H. Arntzen M. O. (2012). MiR-133b targets antiapoptotic genes and enhances death receptor-induced apoptosis. *PLoS One* 7:e35345. 10.1371/journal.pone.0035345 22532850 PMC3332114

[B21] Paul P. Chakraborty A. Sarkar D. Langthasa M. Rahman M. Bari M. (2018). Interplay between miRNAs and human diseases. *J. Cell. Physiol.* 233 2007–2018. 10.1002/jcp.25854 28181241

[B22] Rao X. Huang X. Zhou Z. Lin X. (2013). An improvement of the 2^(-delta delta CT) method for quantitative real-time polymerase chain reaction data analysis. *Biostat. Bioinforma. Biomath.* 3 71–85. 25558171 PMC4280562

[B23] Santosa D. Castoldi M. Paluschinski M. Sommerfeld A. Haussinger D. (2015). Hyperosmotic stress activates the expression of members of the miR-15/107 family and induces downregulation of anti-apoptotic genes in rat liver. *Sci. Rep.* 5:12292. 10.1038/srep12292 PMC4508667 26195352

[B24] Schneider T. J. Fischer G. M. Donohoe T. J. Colarusso T. P. Rothstein T. L. (1999). A novel gene coding for a Fas apoptosis inhibitory molecule (FAIM) isolated from inducibly Fas-resistant B lymphocytes. *J. Exp. Med.* 189 949–956. 10.1084/jem.189.6.949 10075978 PMC2193037

[B25] Segura M. F. Sole C. Pascual M. Moubarak R. S. Perez-Garcia M. J. Gozzelino R. (2007). The long form of Fas apoptotic inhibitory molecule is expressed specifically in neurons and protects them against death receptor-triggered apoptosis. *J. Neurosci.* 27 11228–11241. 10.1523/jneurosci.3462-07.2007 17942717 PMC6673028

[B26] Shingara J. Keiger K. Shelton J. Laosinchai-Wolf W. Powers P. Conrad R. (2005). An optimized isolation and labeling platform for accurate microRNA expression profiling. *RNA* 11 1461–1470. 10.1261/rna.2610405 16043497 PMC1370829

[B27] Tognon R. Gasparotto E. P. Leroy J. M. Oliveira G. L. Neves R. P. Carrara Rde C. (2011). Differential expression of apoptosis-related genes from death receptor pathway in chronic myeloproliferative diseases. *J. Clin. Pathol.* 64 75–82. 10.1136/jcp.2010.080895 21045235

[B28] Vlachos I. S. Zagganas K. Paraskevopoulou M. D. Georgakilas G. Karagkouni D. Vergoulis T. (2015). DIANA-miRPath v3.0: deciphering microRNA function with experimental support. *Nucleic Acids Res.* 43 W460–W466. 25977294 10.1093/nar/gkv403 PMC4489228

[B29] Xie B. Zhou H. Zhang R. Song M. Yu L. Wang L. (2015). Serum miR-206 and miR-132 as potential circulating biomarkers for mild cognitive impairment. *J. Alzheimers. Dis.* 45 721–731. 10.3233/jad-142847 25589731

[B30] Yang Q. Zhao Q. Yin Y. (2019). miR-133b is a potential diagnostic biomarker for Alzheimer’s disease and has a neuroprotective role. *Exp. Ther. Med.* 18 2711–2718. 31572518 10.3892/etm.2019.7855 PMC6755445

[B31] Yu L. Y. Saarma M. Arumae U. (2008). Death receptors and caspases but not mitochondria are activated in the GDNF- or BDNF-deprived dopaminergic neurons. *J. Neurosci.* 28 7467–7475. 10.1523/jneurosci.1877-08.2008 18650325 PMC6670859

[B32] Zhong X. Schneider T. J. Cabral D. S. Donohoe T. J. Rothstein T. L. (2001). An alternatively spliced long form of Fas apoptosis inhibitory molecule (FAIM) with tissue-specific expression in the brain. *Mol. Immunol.* 38 65–72. 10.1016/s0161-5890(01)00035-9 11483211

