# Supplementary figures and images for: FAIM Is Regulated by MiR-206, MiR-1-3p and MiR-133b
Source: Front Cell Dev Biol. 2020 Dec 23;8:584606. doi: 10.3389/fcell.2020.584606 (PMC7785887; doi:10.3389/fcell.2020.584606)

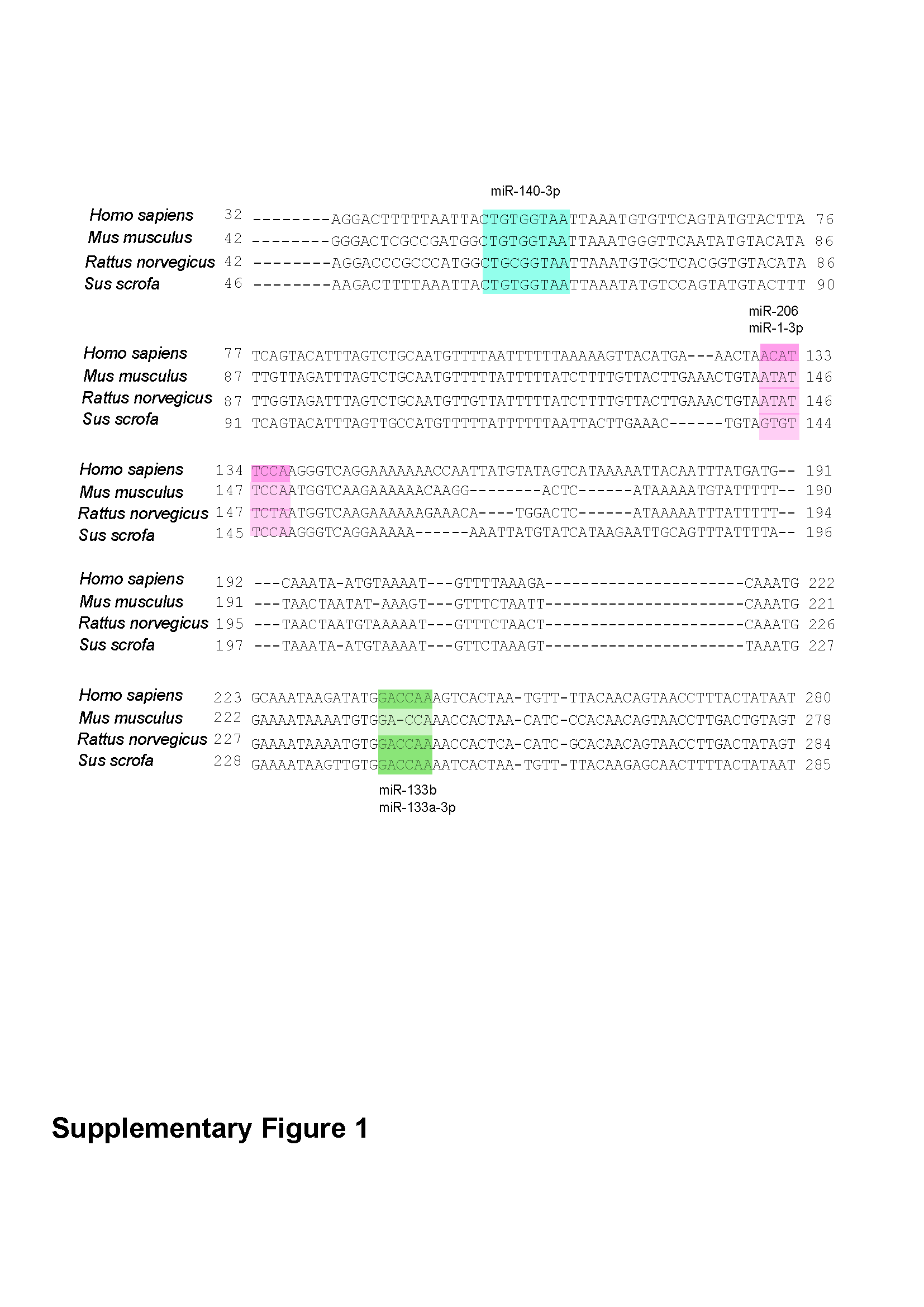

Supplement: Supplementary Figure 1 — Multiple sequence alignment of 3′UTR FAIM from different species. Sequences included in the alignment are those of Homo sapiens, Mus musculus, Rattus norvegicus, and Sus scrofa. The sequences of miRNA are boxed in blue (miR-140-3p), pink (miR-206;miR-1-3p), and green (miR-133b;miR-133a-3p). [file Image_1.TIFF]
